# Supplementary material for: Modulation pattern recognition method of wireless communication automatic system based on IABLN algorithm in intelligent system
Source: PLoS One. 2025 Jan 13;20(1):e0317355. doi: 10.1371/journal.pone.0317355 (PMC11729949; doi:10.1371/journal.pone.0317355)
Supplement: S1 Dataset — (DOC) [file pone.0317355.s001.doc]

**Figure 8 Original data set**

| SNR | Accuracy | | | | | F1-value | | | | |
| --- | --- | --- | --- | --- | --- | --- | --- | --- | --- | --- |
|  | Two layer GRU | One layer LSTM | Two layer LSTM | Bidirectional LSTM | IBLSTM | Two layer GRU | One layer LSTM | Two layer LSTM | Bidirectional LSTM | IBLSTM |
| -20 | 0.10 | 0.10 | 0.10 | 0.10 | 0.10 | 0.10 | 0.10 | 0.10 | 0.10 | 0.10 |
| -16 | 0.09 | 0.10 | 0.09 | 0.10 | 0.13 | 0.11 | 0.11 | 0.12 | 0.13 | 0.15 |
| -12 | 0.12 | 0.12 | 0.12 | 0.15 | 0.25 | 0.20 | 0.20 | 0.20 | 0.21 | 0.23 |
| -8 | 0.33 | 0.35 | 0.36 | 0.40 | 0.46 | 0.52 | 0.54 | 0.57 | 0.60 | 0.61 |
| -4 | 0.62 | 0.63 | 0.65 | 0.68 | 0.74 | 0.79 | 0.80 | 0.80 | 0.81 | 0.81 |
| 0 | 0.84 | 0.85 | 0.85 | 0.90 | 0.91 | 0.87 | 0.89 | 0.90 | 0.90 | 0.91 |
| 4 | 0.87 | 0.87 | 0.90 | 0.91 | 0.92 | 0.90 | 0.91 | 0.93 | 0.93 | 0.94 |
| 8 | 0.85 | 0.87 | 0.90 | 0.91 | 0.92 | 0.91 | 0.93 | 0.93 | 0.93 | 0.95 |
| 12 | 0.87 | 0.89 | 0.90 | 0.92 | 0.93 | 0.93 | 0.93 | 0.93 | 0.93 | 0.95 |
| 16 | 0.87 | 0.90 | 0.91 | 0.91 | 0.92 | 0.91 | 0.93 | 0.93 | 0.93 | 0.95 |

**Figure 9 Original data set**

| SNR | Accuracy | | F1-value | |
| --- | --- | --- | --- | --- |
|  | IBLSTM | IABLN | IBLSTM | IABLN |
| -20 | 0.10 | 0.10 | 0.10 | 0.10 |
| -16 | 0.13 | 0.15 | 0.15 | 0.18 |
| -12 | 0.19 | 0.23 | 0.19 | 0.26 |
| -8 | 0.37 | 0.39 | 0.52 | 0.57 |
| -4 | 0.74 | 0.80 | 0.81 | 0.85 |
| 0 | 0.91 | 0.94 | 0.91 | 0.95 |
| 4 | 0.92 | 0.96 | 0.94 | 0.96 |
| 8 | 0.92 | 0.96 | 0.95 | 0.98 |
| 12 | 0.93 | 0.96 | 0.95 | 0.98 |
| 16 | 0.92 | 0.96 | 0.95 | 0.98 |

**Figure 10 Original data set**

| Performance index | IBLSTM | IABLN |
| --- | --- | --- |
| OA | 0.58 | 0.64 |
| AA | 0.60 | 0.65 |
| MA | 0.90 | 0.93 |
| KC | 0.56 | 0.62 |

**Figure 11 Original data set**

| Output unit number | | | | | | | | | | | | | | | | | | | | |
| --- | --- | --- | --- | --- | --- | --- | --- | --- | --- | --- | --- | --- | --- | --- | --- | --- | --- | --- | --- | --- |
| 1 | 2 | 3 | 4 | 5 | 6 | 7 | 8 | 9 | 10 | 11 | 12 | 13 | 14 | 15 | 16 | 17 | 18 | 19 | 20 | 21 |
| 0.00 | 0.30 | 0.50 | 0.80 | 0.02 | 1.00 | 0.30 | 0.25 | 0.70 | 0.01 | 0.02 | 1.00 | 0.80 | 0.33 | 0.40 | 0.50 | 0.40 | 0.2 | 0.01 | 0.40 | 0.08 |
| Output unit number | | | | | | | | | | | | | | | | | | | | |
| 22 | 23 | 24 | 25 | 26 | 27 | 28 | 29 | 30 | 31 | 32 | 33 | 34 | 35 | 36 | 37 | 38 | 39 | 40 | 41 | 42 |
| 0.42 | 0.20 | 0.12 | 0.18 | 0.28 | 0.40 | 0.01 | 0.00 | 0.60 | 0.00 | 0.00 | 0.20 | 0.00 | 0.12 | 0.30 | 0.70 | 1.00 | 0.01 | 0.85 | 0.20 | 0.00 |

**Figure 12 Original data set**

| SNR=-12dB | | | | | | | | | | |
| --- | --- | --- | --- | --- | --- | --- | --- | --- | --- | --- |
| / | WBFM | QPSK | QAM64 | QAM16 | PAM4 | GFSK | CPFSK | BPSK | AM-DSB | 8PSK |
| WBFM | 0.12 | 0.04 | 0.03 | 0.01 | 0.01 | 0.07 | 0.06 | 0.09 | 0.25 | 0.32 |
| QPSK | 0.02 | 0.03 | 0.02 | 0.04 | 0.03 | 0.06 | 0.03 | 0.03 | 0.55 | 0.19 |
| QAM64 | 0.00 | 0.19 | 0.19 | 0.29 | 0.03 | 0.02 | 0.07 | 0.02 | 0.00 | 0.19 |
| QAM16 | 0.00 | 0.18 | 0.07 | 0.04 | 0.05 | 0.08 | 0.14 | 0.08 | 0.04 | 0.32 |
| PAM4 | 0.03 | 0.05 | 0.06 | 0.02 | 0.10 | 0.14 | 0.05 | 0.10 | 0.22 | 0.23 |
| GFSK | 0.03 | 0.06 | 0.16 | 0.03 | 0.05 | 0.07 | 0.11 | 0.23 | 0.04 | 0.22 |
| CPFSK | 0.08 | 0.03 | 0.14 | 0.13 | 0.04 | 0.04 | 0.05 | 0.11 | 0.13 | 0.25 |
| BPSK | 0.01 | 0.01 | 0.24 | 0.19 | 0.00 | 0.06 | 0.17 | 0.09 | 0.12 | 0.11 |
| AM-DSB | 0.08 | 0.02 | 0.03 | 0.02 | 0.03 | 0.05 | 0.01 | 0.05 | 0.29 | 0.42 |
| 8PSK | 0.11 | 0.02 | 0.02 | 0.04 | 0.03 | 0.04 | 0.01 | 0.01 | 0.47 | 0.25 |
| SNR=0dB | | | | | | | | | | |
| / | WBFM | QPSK | QAM64 | QAM16 | PAM4 | GFSK | CPFSK | BPSK | AM-DSB | 8PSK |
| WBFM | 0.32 | 0.00 | 0.00 | 0.00 | 0.00 | 0.05 | 0.00 | 0.00 | 0.63 | 0.00 |
| QPSK | 0.00 | 0.88 | 0.01 | 0.02 | 0.00 | 0.00 | 0.01 | 0.00 | 0.00 | 0.08 |
| QAM64 | 0.00 | 0.00 | 0.83 | 0.16 | 0.00 | 0.00 | 0.00 | 0.00 | 0.00 | 0.01 |
| QAM16 | 0.00 | 0.00 | 0.11 | 0.84 | 0.01 | 0.00 | 0.00 | 0.02 | 0.00 | 0.02 |
| PAM4 | 0.00 | 0.00 | 0.00 | 0.00 | 0.99 | 0.00 | 0.01 | 0.00 | 0.00 | 0.00 |
| GFSK | 0.01 | 0.00 | 0.00 | 0.00 | 0.00 | 0.99 | 0.00 | 0.00 | 0.00 | 0.00 |
| CPFSK | 0.00 | 0.00 | 0.00 | 0.00 | 0.00 | 0.00 | 1.00 | 0.00 | 0.00 | 0.00 |
| BPSK | 0.00 | 0.00 | 0.00 | 0.01 | 0.03 | 0.00 | 0.00 | 0.96 | 0.00 | 0.00 |
| AM-DSB | 0.11 | 0.00 | 0.00 | 0.00 | 0.00 | 0.00 | 0.00 | 0.00 | 0.89 | 0.00 |
| 8PSK | 0.00 | 0.06 | 0.01 | 0.03 | 0.00 | 0.00 | 0.01 | 0.00 | 0.00 | 0.89 |

**Figure 13 Original data set**

| SNR=6dB | | | | | | | | | | |
| --- | --- | --- | --- | --- | --- | --- | --- | --- | --- | --- |
| / | WBFM | QPSK | QAM64 | QAM16 | PAM4 | GFSK | CPFSK | BPSK | AM-DSB | 8PSK |
| WBFM | 0.44 | 0.00 | 0.00 | 0.00 | 0.00 | 0.00 | 0.00 | 0.00 | 0.56 | 0.00 |
| QPSK | 0.00 | 0.99 | 0.00 | 0.00 | 0.00 | 0.00 | 0.00 | 0.01 | 0.00 | 0.00 |
| QAM64 | 0.00 | 0.00 | 0.83 | 0.17 | 0.00 | 0.00 | 0.00 | 0.00 | 0.00 | 0.00 |
| QAM16 | 0.00 | 0.00 | 0.05 | 0.88 | 0.00 | 0.00 | 0.02 | 0.01 | 0.01 | 0.03 |
| PAM4 | 0.00 | 0.00 | 0.00 | 0.00 | 1.00 | 0.00 | 0.00 | 0.00 | 0.00 | 0.00 |
| GFSK | 0.00 | 0.00 | 0.00 | 0.00 | 0.00 | 1.00 | 0.00 | 0.00 | 0.00 | 0.00 |
| CPFSK | 0.00 | 0.00 | 0.00 | 0.00 | 0.00 | 0.00 | 1.00 | 0.00 | 0.00 | 0.00 |
| BPSK | 0.00 | 0.00 | 0.00 | 0.00 | 0.01 | 0.00 | 0.00 | 0.99 | 0.00 | 0.00 |
| AM-DSB | 0.01 | 0.00 | 0.00 | 0.00 | 0.00 | 0.00 | 0.00 | 0.00 | 0.99 | 0.00 |
| 8PSK | 0.00 | 0.00 | 0.00 | 0.00 | 0.00 | 0.00 | 0.01 | 0.00 | 0.00 | 0.99 |
| SNR=0dB | | | | | | | | | | |
| / | WBFM | QPSK | QAM64 | QAM16 | PAM4 | GFSK | CPFSK | BPSK | AM-DSB | 8PSK |
| WBFM | 0.36 | 0.00 | 0.00 | 0.00 | 0.00 | 0.00 | 0.00 | 0.00 | 0.64 | 0.00 |
| QPSK | 0.00 | 0.99 | 0.00 | 0.00 | 0.00 | 0.00 | 0.00 | 0.00 | 0.00 | 0.01 |
| QAM64 | 0.00 | 0.00 | 0.82 | 0.18 | 0.00 | 0.00 | 0.00 | 0.00 | 0.00 | 0.00 |
| QAM16 | 0.00 | 0.00 | 0.03 | 0.96 | 0.00 | 0.00 | 0.00 | 0.00 | 0.00 | 0.01 |
| PAM4 | 0.00 | 0.00 | 0.00 | 0.00 | 0.98 | 0.00 | 0.00 | 0.00 | 0.00 | 0.02 |
| GFSK | 0.00 | 0.00 | 0.00 | 0.00 | 0.00 | 1.00 | 0.00 | 0.00 | 0.00 | 0.00 |
| CPFSK | 0.00 | 0.00 | 0.00 | 0.00 | 0.00 | 0.00 | 1.00 | 0.00 | 0.00 | 0.00 |
| BPSK | 0.00 | 0.00 | 0.00 | 0.00 | 0.00 | 0.00 | 0.00 | 1.00 | 0.00 | 0.00 |
| AM-DSB | 0.00 | 0.00 | 0.00 | 0.00 | 0.00 | 0.00 | 0.00 | 0.00 | 1.00 | 0.00 |
| 8PSK | 0.00 | 0.01 | 0.00 | 0.00 | 0.00 | 0.00 | 0.00 | 0.00 | 0.00 | 0.99 |

**Figure 14 Original data set**

| SNR | Accuracy | | | | | | F1-value | | | | | |
| --- | --- | --- | --- | --- | --- | --- | --- | --- | --- | --- | --- | --- |
|  | IABLN | LSTM | DesNet | ResNet | Reference[12] | Reference[18] | IABLN | LSTM | DesNet | ResNet | Reference[12] | Reference[18] |
| -20 | 0.10 | 0.10 | 0.10 | 0.10 | 0.10 | 0.10 | 0.10 | 0.10 | 0.10 | 0.10 | 0.10 | 0.10 |
| -18 | 0.12 | 0.10 | 0.10 | 0.10 | 0.12 | 0.09 | 0.11 | 0.10 | 0.10 | 0.10 | 0.10 | 0.11 |
| -16 | 0.15 | 0.11 | 0.12 | 0.12 | 0.10 | 0.14 | 0.14 | 0.11 | 0.11 | 0.11 | 0.11 | 0.12 |
| -14 | 0.18 | 0.17 | 0.17 | 0.15 | 0.12 | 0.18 | 0.17 | 0.14 | 0.13 | 0.13 | 0.13 | 0.15 |
| -12 | 0.22 | 0.17 | 0.17 | 0.17 | 0.13 | 0.19 | 0.24 | 0.22 | 0.22 | 0.22 | 0.20 | 0.22 |
| -10 | 0.30 | 0.23 | 0.23 | 0.28 | 0.17 | 0.28 | 0.33 | 0.30 | 0.30 | 0.30 | 0.27 | 0.30 |
| -8 | 0.48 | 0.37 | 0.37 | 0.41 | 0.25 | 0.37 | 0.48 | 0.40 | 0.42 | 0.45 | 0.38 | 0.46 |
| -6 | 0.61 | 0.50 | 0.50 | 0.52 | 0.38 | 0.50 | 0.63 | 0.57 | 0.58 | 0.59 | 0.51 | 0.61 |
| -4 | 0.77 | 0.61 | 0.60 | 0.70 | 0.53 | 0.61 | 0.78 | 0.68 | 0.70 | 0.70 | 0.62 | 0.74 |
| -2 | 0.87 | 0.78 | 0.77 | 0.80 | 0.60 | 0.78 | 0.90 | 0.80 | 0.81 | 0.83 | 0.73 | 0.86 |
| 0 | 0.90 | 0.80 | 0.86 | 0.88 | 0.69 | 0.85 | 0.92 | 0.88 | 0.90 | 0.90 | 0.78 | 0.90 |
| 2 | 0.92 | 0.85 | 0.88 | 0.90 | 0.70 | 0.90 | 0.93 | 0.90 | 0.90 | 0.90 | 0.80 | 0.91 |
| 4 | 0.93 | 0.86 | 0.89 | 0.92 | 0.70 | 0.93 | 0.93 | 0.90 | 0.90 | 0.90 | 0.80 | 0.91 |
| 6 | 0.93 | 0.85 | 0.89 | 0.92 | 0.70 | 0.92 | 0.93 | 0.90 | 0.90 | 0.90 | 0.80 | 0.91 |
| 8 | 0.93 | 0.85 | 0.89 | 0.91 | 0.70 | 0.91 | 0.93 | 0.90 | 0.90 | 0.90 | 0.80 | 0.91 |
| 10 | 0.93 | 0.85 | 0.89 | 0.91 | 0.70 | 0.90 | 0.93 | 0.90 | 0.90 | 0.90 | 0.80 | 0.91 |
| 12 | 0.93 | 0.85 | 0.89 | 0.91 | 0.72 | 0.90 | 0.93 | 0.90 | 0.90 | 0.90 | 0.81 | 0.91 |
| 14 | 0.93 | 0.86 | 0.90 | 0.91 | 0.73 | 0.90 | 0.93 | 0.90 | 0.90 | 0.90 | 0.81 | 0.91 |
| 16 | 0.93 | 0.85 | 0.89 | 0.91 | 0.74 | 0.90 | 0.93 | 0.90 | 0.90 | 0.90 | 0.81 | 0.91 |
| 18 | 0.93 | 0.85 | 0.89 | 0.91 | 0.74 | 0.90 | 0.93 | 0.90 | 0.90 | 0.90 | 0.81 | 0.91 |
