# Supplementary material for: Modulation pattern recognition method of wireless communication automatic system based on IABLN algorithm in intelligent system
Source: PLoS One. 2025 Jan 13;20(1):e0317355. doi: 10.1371/journal.pone.0317355 (PMC11729949; doi:10.1371/journal.pone.0317355)
Supplement: Attachment — Submitted filename: Response to reviewers comments.doc [file pone.0317355.s002.doc]

Reviewers' comments:

Reviewer's Responses to Questions

Comments to the Author

1. Is the manuscript technically sound, and do the data support the conclusions?

The manuscript must describe a technically sound piece of scientific research with data that supports the conclusions. Experiments must have been conducted rigorously, with appropriate controls, replication, and sample sizes. The conclusions must be drawn appropriately based on the data presented.

Reviewer #1: Partly

Reviewer #2: Partly

Reply: Thank you very much for your comments. The proposed methods, experimental results discussion, conclusion and contribution have been explained in more detail to make the manuscript more technically sound. Please see the revisions as follows:

A two-way interactive temporal network based on LSTM is studied and designed to enhance the contextualization capability of the temporal network. By introducing multiple rounds of interactive operations, the ability to extract modulation pattern information for wireless communication systems is improved. The application of soft attention mechanisms within the IABLSTM network enables the model to prioritize pertinent information pertinent to the current task, thereby enhancing its overall performance. The modulation pattern recognition method for wireless communication automation systems, which is based on the IABLN algorithm developed in the study, addresses the limitations of temporal networks in long input sequences and the lack of temporal information sensitivity in convolutional networks. Overall, the recognition method improves the contextualization capability of the temporal network, speeds up the inference process, and improves the recognition accuracy of the network, which has a good application prospect in the field of wireless communication. The proposed recognition method has the potential to enhance the capabilities of temporal networks in summarizing past and future information, thereby facilitating the advancement of modulation pattern recognition technology for automatic systems. Furthermore, it offers theoretical and technical support for the development of intelligent communications.

Before input $x^{i}$ at the current moment enters the cell of the LSTM, $x^{i}$ is interacted with another input $h_{t-1}$ of the LSTM for multiple rounds, thus enhancing the contextual modeling capability.

where $x_{-1}=x_{t}$, $h_{prev}^{0}=h_{t-1}$, and both denote the current moment's input and the previous moment's output hidden state, respectively.

On this basis, the study further utilizes the CSPB.ML2018 dataset and the RML2016.09a dataset for performance checking. The CSPB.ML2018 dataset is an improvement of the RML2016 10.b dataset, which addresses the known issues and survey errors of the RML2016 dataset. It contains 8 different digital modulation modes, 3584000 signal samples. The RML2016.09a dataset is an earlier version of the RML2016 dataset that also contains signal samples for multiple modulation types. The performance evaluation results of the six methods on the CSPB.ML2018 dataset are shown in Table 3.

**Table 3. Performance evaluation results of the six methods in the CSPB.ML2018 dataset and RML2016.09a dataset**

| Method and datasets | | Total number of parameters | Inference time | OA | AA | KC | *p*-value |
| --- | --- | --- | --- | --- | --- | --- | --- |
| LSTM | CSPB.ML2018 | 220.00k | 30.24ms | 50.55% | 51.02% | 0.5 | 0.001 |
|  | RML2016.09a | 200.00k | 19.87ms | 65.55% | 54.65% | 0.46 |  |
| DesNet | CSPB.ML2018 | 300.00k | 28.56ms | 56.87% | 55.46% | 0.53 | 0.001 |
|  | RML2016.09a | 250.00k | 20.09ms | 61.02% | 60.88% | 0.5 |  |
| ResNet | CSPB.ML2018 | 200.00k | 21.88ms | 64.72% | 63.25% | 0.61 | 0.001 |
|  | RML2016.09a | 180.00k | 15.68ms | 70.75% | 68.48% | 0.64 |  |
| IABLN | CSPB.ML2018 | 300.00k | 4.95ms | 65.07% | 64.21% | 0.62 | - |
|  | RML2016.09a | 280.00k | 3.86ms | 78.65% | 74.21% | 0.68 |  |
| Transformer | CSPB.ML2018 | 250.00k | 4.54ms | 65.60% | 64.33% | 0.64 | 0.098 |
|  | RML2016.09a | 200.00k | 4.01ms | 80.09% | 75.51% | 0.67 |  |
| CNN | CSPB.ML2018 | 240.00k | 22.45ms | 59.78% | 56.54% | 0.53 | 0.003 |
|  | RML2016.09a | 200.00k | 12.33ms | 68.78% | 70.23% | 0.62 |  |

Finally, the study further performs a time complexity analysis. Assuming that the length of the input signal is N, the size of the convolutional kernel is K, the number of input channels is C_in_, and the number of output channels is C_out_. The computational complexity of each output feature map is O(N·C_in_·K), and the total complexity of the convolutional layer is O(C_out_·N·C_in_·K). For each direction of the LSTM, the time complexity is O(N·D). Among them, D is the hidden layer dimension of the LSTM cell. Since the LSTM used in the study is bi-directional, the total time complexity of BiLSTM is O(2·N·D). The attentional mechanism necessitates the scoring and normalization of each element within the sequence, which is of a complexity of O(N·D) for each output time step and a total time complexity of O(N^2^·D). Assuming that the output dimension of the fully connected layer is M, the complexity is O(N·D·M). Combining the above, the total time complexity of the IABLN algorithm proposed in the study is O(N·(C_out_·C_in_·K+3·D+D·M)+N^2^·D).

The computational complexity of the traditional one-way LSTM is less than that of the IABLN, as it is only processed once at each time step. Conversely, CNNs primarily extract features through convolutional and pooling layers, which typically exhibit a reduced computational complexity, particularly when processing high-dimensional data such as images. However, CNNs do not typically address temporal information. The Transformer deals with the data by means of a self-attentive mechanism, which can process all elements in a sequence in parallel. Moreover, the computational complexity is related to the length of the sequence and the number of attention heads. Although Transformer performs well in some tasks, its computational complexity is usually higher than that of LSTM, especially when the sequence length is long. The preceding experimental results demonstrate that the IABLN algorithm exhibits high recognition accuracy and a rapid response time on diverse datasets. These findings suggest that the algorithm is an effective approach for modulation recognition tasks, despite its high computational complexity.

2. Has the statistical analysis been performed appropriately and rigorously?

Reviewer #1: Yes

Reviewer #2: N/A

Reply: Thank you very much for your comments. In order to make the statistical analysis of the experimental results more comprehensive, the results have been supplemented with an analysis of the accuracy and validation results of the other datasets, which can be seen in Table 3. In addition, an analysis of the time complexity of the algorithms has been added, which can be seen in Section 4.3 of the manuscript.

3. Have the authors made all data underlying the findings in their manuscript fully available?

The PLOS Data policy requires authors to make all data underlying the findings described in their manuscript fully available without restriction, with rare exception (please refer to the Data Availability Statement in the manuscript PDF file). The data should be provided as part of the manuscript or its supporting information, or deposited to a public repository. For example, in addition to summary statistics, the data points behind means, medians and variance measures should be available. If there are restrictions on publicly sharing data—e.g. participant privacy or use of data from a third party—those must be specified.

Reviewer #1: Yes

Reviewer #2: Yes

4. Is the manuscript presented in an intelligible fashion and written in standard English?

PLOS ONE does not copyedit accepted manuscripts, so the language in submitted articles must be clear, correct, and unambiguous. Any typographical or grammatical errors should be corrected at revision, so please note any specific errors here.

Reviewer #1: Yes

Reviewer #2: No

Reply: Thank you very much for your comments. The language of the manuscript has been modified by some scholars who have published English papers.

5. Review Comments to the Author

Please use the space provided to explain your answers to the questions above. You may also include additional comments for the author, including concerns about dual publication, research ethics, or publication ethics. (Please upload your review as an attachment if it exceeds 20,000 characters)

Reviewer #1:

(1) The added network model must be analyzed for performance evaluation.

Reply: Thank you very much for your comments. The performance evaluation of the added network model has been analyzed as follows:

Finally, the study further performs a time complexity analysis. Assuming that the length of the input signal is N, the size of the convolutional kernel is K, the number of input channels is C_in_, and the number of output channels is C_out_. The computational complexity of each output feature map is O(N·C_in_·K), and the total complexity of the convolutional layer is O(C_out_·N·C_in_·K). For each direction of the LSTM, the time complexity is O(N·D). Among them, D is the hidden layer dimension of the LSTM cell. Since the LSTM used in the study is bi-directional, the total time complexity of BiLSTM is O(2·N·D). The attentional mechanism necessitates the scoring and normalization of each element within the sequence, which is of a complexity of O(N·D) for each output time step and a total time complexity of O(N^2^·D). Assuming that the output dimension of the fully connected layer is M, the complexity is O(N·D·M). Combining the above, the total time complexity of the IABLN algorithm proposed in the study is O(N·(C_out_·C_in_·K+3·D+D·M)+N^2^·D).

The computational complexity of the traditional one-way LSTM is less than that of the IABLN, as it is only processed once at each time step. Conversely, CNNs primarily extract features through convolutional and pooling layers, which typically exhibit a reduced computational complexity, particularly when processing high-dimensional data such as images. However, CNNs do not typically address temporal information. The Transformer deals with the data by means of a self-attentive mechanism, which can process all elements in a sequence in parallel. Moreover, the computational complexity is related to the length of the sequence and the number of attention heads. Although Transformer performs well in some tasks, its computational complexity is usually higher than that of LSTM, especially when the sequence length is long. The preceding experimental results demonstrate that the IABLN algorithm exhibits high recognition accuracy and a rapid response time on diverse datasets. These findings suggest that the algorithm is an effective approach for modulation recognition tasks, despite its high computational complexity.

(2) The contribution of this work must be clearly stated in the introduction section.

Reply: Thank you very much for your comments. The contributions of this study have been clearly stated in the introduction section. Please see details as follows:

A two-way interactive temporal network based on LSTM is studied and designed to enhance the contextualization capability of the temporal network. By introducing multiple rounds of interactive operations, the ability to extract modulation pattern information for wireless communication systems is improved. The application of soft attention mechanisms within the IABLSTM network enables the model to prioritize pertinent information pertinent to the current task, thereby enhancing its overall performance. The modulation pattern recognition method for wireless communication automation systems, which is based on the IABLN algorithm developed in the study, addresses the limitations of temporal networks in long input sequences and the lack of temporal information sensitivity in convolutional networks. Overall, the recognition method improves the contextualization capability of the temporal network, speeds up the inference process, and improves the recognition accuracy of the network, which has a good application prospect in the field of wireless communication. The proposed recognition method has the potential to enhance the capabilities of temporal networks in summarizing past and future information, thereby facilitating the advancement of modulation pattern recognition technology for automatic systems. Furthermore, it offers theoretical and technical support for the development of intelligent communications.

(3) In the last paragrapgh of section 1, the section number has a problem.

Reply: Thank you very much for your comments. Please see the revisions as follows:

The overall framework of the study can be divided into five sections. In Section 1, a synthesis is presented of the domestic and international achievements and shortcomings pertaining to modulation pattern recognition methods for the development of automatic wireless communication systems. In Section 2, the study proposes the IABLN algorithm and based on this, the design of modulation pattern recognition methods based on the IABLN algorithm is carried out. In Section 3, the experimental simulation and analysis are carried out. In the fourth part, the experimental results are discussed and analyzed. In Section 4, the research findings are summarized and directions for further research are pointed out.

(4) The computational complexity of the proposed method should be analyzed to compared with the other representative methods.

Reply: Thank you very much for your valuable suggestions. The computational complexity of the proposed method has been analyzed to compared with the other representative methods. Please see details as follows:

Finally, the study further performs a time complexity analysis. Assuming that the length of the input signal is N, the size of the convolutional kernel is K, the number of input channels is C_in_, and the number of output channels is C_out_. The computational complexity of each output feature map is O(N·C_in_·K), and the total complexity of the convolutional layer is O(C_out_·N·C_in_·K). For each direction of the LSTM, the time complexity is O(N·D). Among them, D is the hidden layer dimension of the LSTM cell. Since the LSTM used in the study is bi-directional, the total time complexity of BiLSTM is O(2·N·D). The attentional mechanism necessitates the scoring and normalization of each element within the sequence, which is of a complexity of O(N·D) for each output time step and a total time complexity of O(N^2^·D). Assuming that the output dimension of the fully connected layer is M, the complexity is O(N·D·M). Combining the above, the total time complexity of the IABLN algorithm proposed in the study is O(N·(C_out_·C_in_·K+3·D+D·M)+N^2^·D).

The computational complexity of the traditional one-way LSTM is less than that of the IABLN, as it is only processed once at each time step. Conversely, CNNs primarily extract features through convolutional and pooling layers, which typically exhibit a reduced computational complexity, particularly when processing high-dimensional data such as images. However, CNNs do not typically address temporal information. The Transformer deals with the data by means of a self-attentive mechanism, which can process all elements in a sequence in parallel. Moreover, the computational complexity is related to the length of the sequence and the number of attention heads. Although Transformer performs well in some tasks, its computational complexity is usually higher than that of LSTM, especially when the sequence length is long. The preceding experimental results demonstrate that the IABLN algorithm exhibits high recognition accuracy and a rapid response time on diverse datasets. These findings suggest that the algorithm is an effective approach for modulation recognition tasks, despite its high computational complexity.

(5) Use different datasets to verify your method.

Reply: Thank you very much for your comments. Different datasets including RML2016 10.b dataset, the RML2016.09a dataset, and the CSPB.ML2018 dataset have been used to verify the proposed method in this study.

Reviewer #2:

1. Clarify your writing, like IABLN in the first time, like the specific version of the dataset.

Reply: Thank you very much for your comments. The full name of “IABLN” in the manuscript is “Interactive Attention Bi-LSTM Network”. The datasets that used in this study are Radio Machine Learning (RML) 2016.10b dataset, RML2016.09a dataset, and Communication Signal Processing Benchmark for Machine Learning (CSPB. ML2018) 2018 dataset.

2. Focus on introduce and elaborate the interactive operation part further.

Reply: Thank you very much for your comments. The information of the interactive operation has been added explained as follows:

Before input $x^{i}$ at the current moment enters the cell of the LSTM, $x^{i}$ is interacted with another input $h_{t-1}$ of the LSTM for multiple rounds, thus enhancing the contextual modeling capability.

where $x_{-1}=x_{t}$, $h_{prev}^{0}=h_{t-1}$, and both denote the current moment's input and the previous moment's output hidden state, respectively.

3. The comparison with state-of-the-art methods needs to be improved.

Reply: Thank you very much for your comments. The comparison with state-of-the-art methods has been improved. Please see details as follows:

To further illustrate the superiority of the IABLN algorithm, the study introduces the current commonly used modulation pattern recognition methods, as well as references [12] and [18], for comparison with IABLN. Meanwhile, the study employs the optimal parameter settings identified by the literature's authors as the relevant parameter settings for the comparison methods. First, the study compares the recognition accuracy and F1 value of the six methods in the RML2016 10.b dataset. The specific comparison results are shown in Figure 14.

On this basis, the study further utilizes the CSPB.ML2018 dataset and the RML2016.09a dataset for performance checking. The CSPB.ML2018 dataset is an improvement of the RML2016 10.b dataset, which addresses the known issues and survey errors of the RML2016 dataset. It contains 8 different digital modulation modes, 3584000 signal samples. The RML2016.09a dataset is an earlier version of the RML2016 dataset that also contains signal samples for multiple modulation types. The performance evaluation results of the six methods on the CSPB.ML2018 dataset are shown in Table 3.

6. PLOS authors have the option to publish the peer review history of their article (what does this mean?). If published, this will include your full peer review and any attached files.

If you choose “no”, your identity will remain anonymous but your review may still be made public.

Do you want your identity to be public for this peer review? For information about this choice, including consent withdrawal, please see our Privacy Policy.

Reviewer #1: No

Reviewer #2: No
