# Supplementary material for: Modulation pattern recognition method of wireless communication automatic system based on IABLN algorithm in intelligent system
Source: PLoS One. 2025 Jan 13;20(1):e0317355. doi: 10.1371/journal.pone.0317355 (PMC11729949; doi:10.1371/journal.pone.0317355)
Supplement: Attachment — Submitted filename: Response to comments.docx [file pone.0317355.s003.docx]

Review Comments to the Author

Reviewer #1: The authors have completed the paper revisions according to the reviewers' comments. It can be accepted for publication.

Reply: Thank you very much for your valuable recommendation (accept).

Reviewer #2: Justify the rationals of the proposed method, i.e., what is the temporal characteristics of modulation data? (not to illustrate with voice data like in the paper).

Reply: Thank you very much for your comments. The temporal characteristics of modulated signals exhibit unique waveform features and changing patterns in time.The IABLN algorithm reinforces the comprehensive capture of past and future information of the signals through bidirectional LSTM, while focusing on the key temporal features by using the attention mechanism. This method can effectively identify the time-varying features and long-term dependencies of modulated signals, thus improving the identification accuracy. Therefore, the algorithm is particularly suitable for processing modulated signals with complex timing characteristics.
